# Evaluation of RNA quality and functional transcriptome of beef *longissimus thoracis* over time *post-mortem*

**DOI:** 10.1371/journal.pone.0251868

**Published:** 2021-05-25

**Authors:** Stephanie Lam, Arun Kommadath, Óscar López-Campos, Nuria Prieto, Jennifer Aalhus, Manuel Juárez, Michael E. R. Dugan, Payam Vahmani

**Affiliations:** 1 Lacombe Research and Development Centre, Agriculture and Agri-Food Canada, Lacombe, Alberta, Canada; 2 Department of Animal Science, University of California Davis, Davis, California, United States of America; INIA, SPAIN

## Abstract

Evaluating RNA quality and transcriptomic profile of beef muscle over time *post-mortem* may provide insight into RNA degradation and underlying biological and functional mechanisms that accompany biochemical changes occurring *post-mortem* during transformation of muscle to meat. RNA was extracted from *longissimus thoracis* (*LT*) sampled from British Continental crossbred heifer carcasses (n = 7) stored at 4°C in an abattoir drip cooler at 5 time points *post-mortem*, i.e., 45 min (0 h), 6 h, 24 h, 48 h, and 72 h. Following RNA-Sequencing, processed reads were aligned to the ARS-UCD1.2 bovine genome assembly. Subsequent differential expression (DE) analysis identified from 51 to 1434 upregulated and 27 to 2256 downregulated DE genes at individual time points compared to time 0 h, showing a trend for increasing counts of both upregulated and downregulated genes over time. Gene ontology and biological pathway term enrichment analyses on sets of DE genes revealed several processes and their timelines of activation/deactivation that accompanied or were involved with muscle transformation to meat. Although the quality of RNA in refrigerated *LT* remained high for several days *post-mortem*, the expression levels of several known biomarker genes for meat quality began to change from 24 h onwards. Therefore, to ensure accuracy of predictions on meat quality traits based on the expression levels of those biomarker genes in refrigerated beef muscle tissue, it is crucial that those expression measurements be made on RNA sampled within 24 h *post-mortem*. The present study also highlighted the need for more research on the roles of mitochondrial genes and non-coding genes in orchestrating muscle tissue processes after death, and how *pre-mortem* immune status might influence *post-mortem* meat quality.

## Introduction

RNA-Sequencing (RNA-Seq) has many applications, some of which include identification of differentially expressed (DE) genes and functional variants, *de novo* transcriptome assembly and novel transcript discovery. The use of RNA-Seq for gene expression analysis relies on careful selection of experimental conditions, including sampling time, physiological condition of individuals sampled, environmental factors, biological tissue, tissue sampling location, as well as sample storage conditions. An important requirement in order to guarantee an accurate snapshot of gene expression at the immediate moment of tissue sampling is the availability of high quality RNA [1]. Since RNA degrades quickly, care should be taken to not only obtain high quality RNA at extraction but also to maintain its quality under appropriate storage conditions until further analysis. Obtaining high quality RNA is especially challenging from *post-mortem* tissue where RNA extraction may either not be possible immediately after organismal death or where RNA samples are required at extended time points *post-mortem*. In addition, initial RNA quality and RNA degradation rates are tissue type dependent. For example, it has been found in beef carcasses stored at 4°C that RNA sampled from skeletal muscle remained at high quality for longer durations *post-mortem* than RNA from liver and adipose tissue [2]. A comparison of multiple *post-mortem* human tissues also indicated RNA from muscle to be amongst the slowest to degrade [3]. Differences in RNA degradation rates have been attributed in part to the concentration of ribonucleases already present in cells and/or originating from bacteria or other environmental contamination, with ribonuclease-rich organs such as pancreas and liver exhibiting quicker RNA fragmentation than others [4].

Studies on *post-mortem* tissues have helped improve the understanding of biological processes and transcriptional changes that continue to occur as the cells shut down after organismal death, with applications such as in forensics to predict time of death [3, 5]. In livestock species, studies on *post-mortem* muscle transcriptomes have helped improve our understanding of the molecular and biological processes that are concurrent with the biochemical changes that occur *post-mortem* as muscle transforms to meat and subsequent aging (e.g. pH, proteolysis, and tenderization), and how those processes influence economically important meat quality characteristics like meat colour and tenderness [2, 6–8]. Recent studies on muscle transcriptome profiles from cattle and pigs have identified genes influencing meat quality traits such as tenderness and marbling in beef and pork [9–12]. The potential for drawing valid inferences between gene biomarkers and meat quality traits at the abattoir could, however, be improved through a better understanding of RNA viability *post-mortem* and expression of specific biomarker genes.

The aims of this study were to improve our understanding of how RNA quality and transcriptomic profiles change in beef muscle over time *post-mortem* under refrigerated abattoir conditions. The specific objectives were to: 1) determine RNA quality in beef *longissimus thoracis* (*LT*) at 5 time points *post-mortem* (0 to 72 h); 2) concurrently evaluate transcriptomic profiles using RNA-Seq to identify DE genes and stability of known biomarker genes for meat quality traits; and 3) identify important biological processes and pathways represented by genes found DE at those time points.

## Materials and methods

### Animals and post-mortem tissue sampling

Experimental conditions were approved by the Agriculture and Agri-Food Canada Lacombe Research and Development Centre (AAFC-Lacombe RDC) Animal Care committee (approval #201705) in compliance with the principles and guidelines established by the Canadian Council on Animal Care (CCAC 2009). This study used 7 (British × Continental crossbred) beef heifers raised at the AAFC-Lacombe RDC farm (Lacombe, AB, Canada) under similar conditions and finished on a barley grain-based diet as described previously [13]. At slaughter, heifers were on average, 567.9 ± 12.5 d old and weighed 574.9 ± 37.0 kg. Animals were slaughtered at the AAFC-Lacombe RDC federally inspected research abattoir using penetrative captive bolt stunning followed by exsanguination. Core *LT* samples were collected at 5 time points *post-mortem*, i.e., 45 min (0 h), 6 h, 24 h, 48 h, and 72 h, from the left carcass sides (stored in the drip cooler at 4°C) above the grading site, and immediately frozen in liquid nitrogen and stored at -80°C until RNA extraction.

### RNA extraction, quality assessment and sequencing

Muscle samples were homogenized using a Tissuelyzer (Qiagen, Valencia, CA) in the presence of Trizol reagent. The RNA was extracted using Qiagen RNAeasy mini kit (Qiagen N.V., Valenca, CA, United States) according to the manufacturer’s protocol. The RNA sample purity was evaluated by determining A260/230 nm and A260/280 nm ratios using the NanoDrop 1000 Spectrophotometer (Thermo Fisher Scientific, Santa Clara, CA, USA, 2007). The RNA sample integrity was assessed using the Agilent 2100 Bioanalyzer (Agilent, Santa Clara, CA, USA) to obtain the RNA integrity number (RIN) values. A total of 35 RNA samples (7 animals × 5 time points) were sequenced at the Génome Québec Innovation Centre (McGill University, Montréal, QC, Canada). Briefly, pooled libraries were prepared and loaded at 225 pM on an Illumina NovaSeq 6000 S4 lane following manufacturer’s protocol. The sequencing run was performed for 2x100 cycles in paired-end mode, generating 100 bp length, paired end sequencing reads. Base calling was performed with RTA v3.4.4 program, and the samples were de-multiplexed to generate sample-wise raw sequence files in fastq format using bcl2fastq2 Conversion Software v2.20.

### RNA-Seq analysis

The quality of sequence data was assessed with FastQC (https://www.bioinformatics.babraham.ac.uk/projects/fastqc/) prior to and after performing quality control steps that included quality based read trimming and adapter removal using Trimmomatic v0.39 [14] with the following parameters: ILLUMINACLIP:/adaptors.fa:2:30:10 LEADING:3 TRAILING:3 SLIDINGWINDOW:4:15 MINLEN:75, where adaptors.fa is a FASTA file containing the oligonucleotide sequences of the Illumina Novaseq adapters used in NEBNext mRNA stranded library preparation kits (New England Biolabs, Ipswich, MA, USA). Reads that passed quality control were mapped to the ARS-UCD1.2 bovine genome assembly [15] using STAR v2.7.0f [16] with default parameters and quantMode set to GeneCounts. Read counts per gene were obtained using featureCounts v1.6.4 [17] in strand specific mode for all genes in the gene annotation file corresponding to the ARS-UCD1.2 bovine genome assembly (from Ensembl [18] release 95) with the following parameters: -s 0 -p -t exon -g gene_id -a Bos_taurus.ARS-UCD1.2.95.gtf. The raw sequence data and read count matrix from this study have been deposited in NCBI’s Gene Expression Omnibus (GEO) database [19] under GEO series accession GSE163766.

### Differential expression analysis

The DE analysis and associated tests were performed in R (R Version 3.6.0.; R Core Team, 2020) statistical programming language using mainly the Bioconductor package, edgeR (version 3.24.3) [20, 21] on read counts from the sense strand. Genes with very low expression were filtered out, keeping only those that were expressed at counts per million (CPM) values that corresponded to a read count over 10 in at least 7 samples (using *filterByExpr* function from edgeR package). Trimmed mean of M-values (TMM) normalization [22] was applied to this dataset to account for compositional differences between the libraries. For exploratory analysis, a principal component analysis (PCA) was performed and plotted using the *plotPCA* function of DESeq2 package (version 1.26.0) [23]. A power analysis was performed using Bioconductor package *RNASeqPower* (version 1.26.0) [24] to determine the appropriate fold change (FC) level that can be reliably detected for this dataset, given the biological co-efficient of variability (BCV) and coverage (read depth) of samples. A negative binomial generalized linear model was used to test for differential gene expression at different time points *post-mortem* compared to the initial time, adding a blocking factor to account for repeated measures over time from the same animal.

### Functional enrichment analysis of differentially expressed genes

Functional enrichment analyses to identify over-represented gene ontology (GO) and Kyoto Encyclopedia of Genes and Genomes (KEGG) biological pathway terms in separate sets of upregulated and downregulated DE genes at different time points were performed using the functional annotation tool from Database for Annotation, Visualization and Integrated Discovery (DAVID) v6.8 database [25, 26]. Ensembl gene IDs were used as the gene identifier and significantly enriched terms (Benjamin-Hochberg corrected *p*-value < 0.05) were determined against a background or population set consisting of all genes identified as expressed, following filtering out of low expressed genes as described in the previous section.

## Results

### RNA quality and RNA-Seq statistics

The RIN values of RNA samples ranged from 7.9 to 8.6, with an average of 8.2 across all sampling time points. The average RIN values across samples for each *post-mortem* sampling time point were 8.3 (0 h), 8.3 (6 h), 8.3 (24 h), 8.1 (48 h), and 8.1 (72 h), with the average dropping slightly after 24 h *post-mortem* (Table 1).

**Table 1:** Animal and sampling information, RIN and RNA-Seq statistics.

Animal ID	Slaughter age (d)	Slaughter weight (kg)	Sampling time^i^	RIN	Total sequenced reads (million)	Uniquely mapped reads (%)
101	581	587	0 h	8.3	85.76	96.00
			6 h	8.0	44.37	95.60
			24 h	8.3	33.12	95.40
			48 h	7.9	114.39	95.70
			72 h	8.0	138.24	95.70
103	549	635	0 h	8.6	47.42	95.40
			6 h	8.1	85.90	95.80
			24 h	8.5	50.81	95.90
			48 h	8.0	38.52	95.80
			72 h	8.0	120.51	95.40
104	579	586	0 h	8.0	41.47	96.50
			6 h	8.5	78.16	95.90
			24 h	8.3	118.08	95.80
			48 h	8.3	50.26	95.80
			72 h	8.1	55.41	95.90
107	549	540	0 h	8.5	71.61	95.90
			6 h	8.6	33.50	96.10
			24 h	8.3	45.66	96.10
			48 h	8.1	35.69	95.80
			72 h	8.1	45.30	95.90
201	575	525	0 h	8.1	85.38	96.00
			6 h	8.0	51.79	95.80
			24 h	8.3	123.09	95.60
			48 h	8.1	54.13	95.90
			72 h	7.9	31.70	95.60
202	571	558	0 h	8.3	28.34	96.00
			6 h	8.4	27.89	95.70
			24 h	8.3	103.32	96.00
			48 h	8.4	73.27	95.70
			72 h	8.2	92.17	95.30
206	553	593	0 h	8.4	84.93	96.20
			6 h	8.5	59.82	95.50
			24 h	8.4	29.69	95.50
			48 h	8.1	28.22	95.60
			72 h	8.1	33.76	95.80

^i^ Sampling time *post-mortem*: 0 h = 45 min.

The number of reads sequenced (in millions) ranged from 27.89 to 138.24 with an average of 64.05 (s.d. 32.12) million reads, of which 95.79% (s.d. 0.25) were uniquely mapped to the bovine reference genome (Table 1 and S1 Fig). The total number of reads post quality control that were assigned to the sense strand ranged from 20.57 to 104.28 million.

### Differentially expressed genes and their characteristics

Read counts were obtained for a total of 27,607 genes annotated in the gene annotation file corresponding to the ARS-UCD1.2 bovine genome assembly. Of those, 14,630 genes (52.99% of the total) were identified as expressed in the *LT* samples used in this experiment, following filtering out of low expressed genes. A PCA plot based on the normalised read counts showed samples separated by time point along the first principal component (PC1) with separation becoming more distinct towards later time points, indicative of substantial and progressively increasing changes in gene expression with time *post-mortem* (S2 Fig). A power analysis suggested that a fold change of 1.5 could be reliably detected at a power of 0.9 and false positive rate of 0.05 with the sample size of 7 per group, and accounting for the average sequencing depth and biological variation within the groups (S3 Fig). At the selected fold change of above 1.5 and a False Discovery Rate (FDR) adjusted *p-*value of below 0.05, the number of DE genes identified showed a steady increase with sampling time *post-mortem*, ranging from 51 to 1434 for upregulated, and 27 to 2256, for downregulated genes, over 4 time points (Table 2). The number of upregulated genes was slightly higher than the downregulated at 6 h but the trend reversed for later time points, and both the number and rate of increase for downregulated genes were much higher. All sets of DE genes are listed in S1 Table and illustrated through heat maps (S4 Fig) and volcano plots (S5 Fig).

**Table 2:** Number of DE genes found in beef *LT* muscle over time.

Sampling time *post-mortem*	Upregulated	Downregulated	Total
**6 h**	51	27	**78**
**24 h**	106	139	**245**
**48 h**	433	922	**1355**
**72 h**	1434	2256	**3690**

Looking into the origin of the DE genes, it was observed that a disproportionately high number of mitochondrial genes were downregulated, and none upregulated at early time points. Mitochondrial genes accounted for below 0.18% (n = 26) of the total 14,630 genes identified as expressed; however, 44.44% of the downregulated genes at 6h (i.e. 12 of 27) and 7.90% of the downregulated genes at 24 h (i.e., 11 of 139) were of mitochondrial origin.

Next, looking at the distribution of gene biotypes among the lists of DE genes, a disproportionately high number of non-coding genes, especially small nucleolar RNA (snoRNA) and long non-coding RNA (lncRNA), appear in both the up and the downregulated sets of genes at all time points. Non-coding genes accounted for below 4% (n = 578) of the total 14,630 genes identified as expressed; however, 33.33% (26 of 78), 17.55% (43 of 245), 9.52% (129 of 1355) and 6.42% (237 of 3690) of the DE genes at 6, 24, 48 and 72 h, respectively, were non-coding.

**Fig 1:**
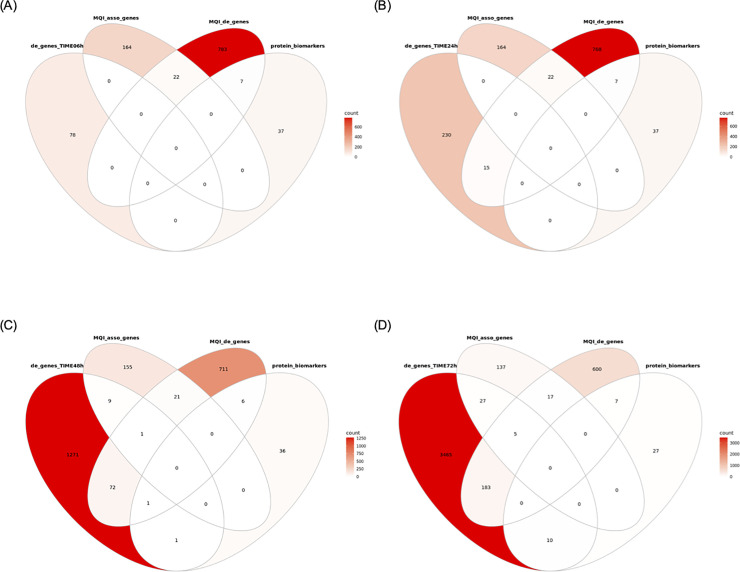
Comparison of previously reported potential biomarker genes for meat quality traits with genes found DE over time in beef *LT* muscle. Panels A-D represent Venn diagrams comparing genes found DE in beef *LT* muscle (at 6, 24, 48 and 72 h, respectively) with previously reported meat quality index (MQI) associated genes (MQI_associated_genes) or DE genes related to MQI traits (MQI_de_genes) or genes encoding protein biomarkers associated with meat quality traits in beef (protein_biomarkers).

Finally, we compared the sets of DE genes found at each time point *post-mortem* with *longissimus dorsi* muscle expressed genes reported recently to be either associated with a meat quality index (MQI; pseudo-phenotype defined through a principal component analysis of several meat quality related traits that included marbling, Warner-Bratzler Shear Force (WBSF), cooking loss, juiciness, tenderness, and connective tissue) or DE between multi-breed Angus-Brahman steers of low or high MQI [27]. In this previous study, an association analysis between gene expression in *longissimus dorsi* muscle from 80 steers with corresponding MQI revealed 208 genes significantly associated with MQI. Of the 208 gene names reported in that study, which were based on an older bovine reference genome annotation, 186 could be converted into Ensembl gene IDs from the current annotation. Remarkably, none of those 186 MQI associated gene IDs were found DE at early time points *post-mortem* (6 and 24 h) in the present study (Fig 1A and 1B). Further, a DE analysis carried out in the previous study between 40 steers with low or high MQI revealed a total of 676, 70 and 198 genes as DE for WBSF, tenderness and marbling, respectively. Combining the 3 sets of DE genes from the previous study resulted in 886 unique gene symbols, of which 812 could be converted to Ensembl gene IDs from the current annotation. Similar to the observation on MQI associated genes, none of those 812 MQI related DE genes were found DE at 6 h post-mortem in the present study (Fig 1A) and only 15 were found DE at 24 h (Fig 1B). The number of genes in common amongst different gene sets increased over time beyond 24 h (Fig 1C and 1D). Next, we made a similar comparison of our combined set of DE genes with a set of 44 Ensembl gene IDs (7 of which were listed in the MQI related DE genes and none in MQI associated genes) of genes that encode the protein biomarkers listed in a recent review [28] as associated with meat quality traits in beef. We found that none of those 44 genes were found DE at 6 h and 12 h, 2 were found DE at 48 h and 10 at 72 h *post-mortem* (Fig 1A–1D). These findings indicate that many potential gene biomarkers associated with meat quality traits have stable gene expression for up to 24 h *post-mortem*.

### Biological processes represented in sets of differentially expressed genes

GO biological processes and KEGG pathway terms found enriched in sets of DE genes from beef *LT* indicated an initial downregulation of oxidative phosphorylation at 6 h, and a surprising reversal to upregulation at 72 h (Table 3). The 8 genes associated with the downregulation of that pathway at 6 h (S2 Table) were completely different from the 36 genes involved in the upregulation of the same process at 72 h. Surprisingly, the sets of upregulated genes did not reveal any significantly enriched processes or pathways at early time-points; however, many immune related terms were found enriched at 48 h, and terms related to ribosome and translation were enriched at 72 h. On the contrary, all 4 sets of downregulated genes indicated several biological processes and pathways to be enriched, progressing from oxidative phosphorylation at 6 h to “regulation of lipolysis in adipocytes” and a few signaling pathways at 24 and 48 h, to a deluge of multiple signaling pathways by 72 h. A detailed overview of all enriched terms from all functional annotation categories tested in DAVID as well as the DE genes annotated to those categories are provided in S2 Table.

**Table 3:** Gene ontology biological process and KEGG pathway terms associated with significantly upregulated and downregulated gene sets in beef *LT* muscle.

Category	Term	Count	List Total	Pop Hits	Pop Total	Fold Enrich ment	BH p-value
***Upregulated at 6 h***	*No terms enriched*						
***Downregulated at 6 h***							
KEGG pathway	bta00190:Oxidative phosphorylation	8	10	130	4595	28.28	2.12E-09
KEGG pathway	bta05012:Parkinson’s disease	8	16	82	11991	63.98	4.05E-09
GO biological process	GO:0042773~ATP synthesis coupled electron transport	3	15	3	11385	759.00	1.39E-04
KEGG pathway	bta01100:Metabolic pathways	8	10	930	4595	3.95	1.11E-03
***Upregulated at 24 h***	*No terms enriched*						
***Downregulated at 24 h***							
KEGG pathway	bta04923:Regulation of lipolysis in adipocytes	8	64	47	4595	12.22	3.39E-04
KEGG pathway	bta04152:AMPK signaling pathway	8	97	14	10576	31.15	2.21E-02
GO biological process	GO:0006094~gluconeogenesis	4	64	106	4595	5.42	3.43E-02
KEGG pathway	bta01100:Metabolic pathways	25	97	775	10576	2.39	3.44E-02
GO biological process	GO:0042773~ATP synthesis coupled electron transport	3	105	159	11991	5.03	4.93E-02
***Upregulated at 48 h***							
KEGG pathway	bta05340:Primary immunodeficiency	8	227	120	10576	5.44	3.41E-04
GO biological process	GO:0070098~chemokine-mediated signaling pathway	8	139	7	6113	31.41	8.75E-04
GO biological process	GO:0007186~G-protein coupled receptor signaling pathway	11	139	22	6113	11.99	5.57E-03
GO biological process	GO:0030593~neutrophil chemotaxis	7	139	206	6113	3.20	7.24E-03
GO biological process	GO:0071346~cellular response to interferon-gamma	6	186	16	8883	14.92	2.25E-02
GO biological process	GO:0048247~lymphocyte chemotaxis	5	110	136	4595	3.69	2.41E-02
KEGG pathway	bta04062:Chemokine signaling pathway	12	208	35	9683	9.31	2.62E-02
KEGG pathway	bta04650:Natural killer cell mediated cytotoxicity	9	110	51	4595	5.73	2.87E-02
KEGG pathway	bta04640:Hematopoietic cell lineage	8	99	5	4423	35.74	3.06E-02
***Downregulated at 48 h***							
KEGG pathway	bta05150:Staphylococcus aureus infection	11	303	48	4595	3.79	1.63E-02
KEGG pathway	bta04923:Regulation of lipolysis in adipocytes	12	303	51	4595	3.57	2.16E-02
KEGG pathway	bta03320:PPAR signaling pathway	12	720	199	11991	2.18	2.48E-02
GO biological process	GO:0009791~post-embryonic development	12	720	3237	11991	1.20	2.90E-02
***Upregulated at 72 h***							
KEGG pathway	bta03010:Ribosome	69	415	119	4595	6.42	2.26E-39
GO biological process	GO:0006412~translation	62	802	148	9683	5.06	5.20E-25
KEGG pathway	bta00190:Oxidative phosphorylation	36	415	121	4595	3.29	1.70E-08
KEGG pathway	bta05016:Huntington’s disease	36	415	130	4595	2.73	1.43E-05
KEGG pathway	bta05012:Parkinson’s disease	32	415	157	4595	2.54	1.86E-05
KEGG pathway	bta05010:Alzheimer’s disease	32	415	149	4595	2.38	2.64E-04
KEGG pathway	bta04932:Non-alcoholic fatty liver disease (NAFLD)	29	415	134	4595	2.40	5.79E-04
KEGG pathway	bta04260:Cardiac muscle contraction	17	1035	589	11991	1.59	8.61E-04
GO biological process	GO:0002181~cytoplasmic translation	10	1035	19	11991	5.49	2.90E-03
***Downregulated at 72 h***							
KEGG pathway	bta05150:Staphylococcus aureus infection	18	664	36	4595	3.46	6.59E-04
KEGG pathway	bta04610:Complement and coagulation cascades	18	664	38	4595	3.28	8.30E-04
KEGG pathway	bta04910:Insulin signaling pathway	34	664	114	4595	2.06	3.47E-03
KEGG pathway	bta04152:AMPK signaling pathway	31	664	106	4595	2.02	7.32E-03
KEGG pathway	bta04068:FoxO signaling pathway	32	664	110	4595	2.01	7.74E-03
KEGG pathway	bta04151:PI3K-Akt signaling pathway	58	664	255	4595	1.57	1.37E-02
KEGG pathway	bta04550:Signaling pathways regulating pluripotency of stem cells	27	664	94	4595	1.99	2.12E-02

## Discussion

Sampling tissues from animals immediately after slaughter is challenging due to limited accessibility and delays when working alongside routine operations in an abattoir. Delays in sampling can affect the quality of RNA extracted from *post-mortem* tissues, which in turn can affect the accuracy of gene expression measurements. However, the storage of carcasses under refrigerated conditions in coolers results in lower enzyme activity, thereby slowing down biochemical processes following death [29] and effectively slowing RNA degradation. In addition to sampling time after slaughter and storage temperature, variability in RNA degradation and quality is dependent on factors such as species and type of tissue sampled [2, 3, 8, 30–33]. For example, a study on RNA quality of pork *semimembranosus* stored at 4°C revealed an average RIN of 3.95 at 48 h [30]. The significantly lower RIN reported in pork compared to the average RIN of 8.1 we obtained for beef *LT* at 48 h may be attributed to the microstructural and cellular differences between pork and beef tissue. This may be due to the variation in muscle fibre type composition across species [34] and the known differences in total RNA content by fibre type in skeletal muscle, in which the slow fiber type (Type I fibre) contains higher RNA content due to higher myonuclei per mm of myofiber compared to fast fiber types (Type II fibres) [35]. Furthermore, RNA degradation rates may be different across species and tissue [2, 3]; in fact, intact RNA has been recovered from beef skeletal muscle for up to 8 d *post-mortem* [2]. Given our confirmation that high quality RNA can be obtained for extended periods in *post-mortem LT* under refrigeration, it was then important to follow-up and determine the duration *post-mortem* that the expression of gene biomarkers for beef quality remained stable. Based on the premise that such biomarkers would be invalid if their expression were to change with time after death, we compared known biomarkers associated with meat quality traits for their presence in our lists of DE genes at different time points *post-mortem*. We found that expression of most biomarkers tested remained stable for up to 24 h *post-mortem* in *LT* muscle stored at 4°C. This finding has important implications when devising sampling regimes for skeletal muscle RNA from the abattoir. While RNA quality is assured for several days *post-mortem*, it is prudent to use samples collected within the first 24 h *post-mortem* when the intent is to predict meat quality based on gene biomarkers associated with meat quality traits.

The increasing number of DE genes detected in this study with progressive time points compared to just after slaughter is indicative of continuing transcriptional activity for extended times after death. While the increase in number of downregulated genes with time *post-mortem* may be intuitively attributed to biological processes shutting down in dying cells, concurrent and substantial increases in numbers of upregulated genes with time came as a surprise. This observation is, however, supported by several studies that reported upregulation of thousands of genes, dependent on time *post-mortem*, tissue type and storage conditions [3, 5, 36]. The sets of upregulated genes were conspicuous by their absence of any enriched biological processes represented in those gene sets during the initial time points. This may suggest that a majority of the genes upregulated at early *post-mortem* were not co-regulated to work in specific pathways or processes. Another possibility is that those genes that would have been part of any processes working in concert may not have been upregulated to the levels required to be detected as significant, given the sample size and statistical power of the current analysis. At 48 h *post-mortem*, many immune related pathways and processes were found upregulated, an observation that has been reported in earlier studies, but at differing time points. For example, transcripts related to immune response increased at 1 and 12 h in mouse brain tissue and transcripts related to immune response in both adaptive and innate immune pathways increased across various time points from 1 h to 24 h in fish liver tissue [5]. Immune and inflammatory responses may be attributed to recognition by the innate immune system of intracellular molecules exposed by dying cells and subsequent macrophage stimulation [37, 38]. The KEGG pathway, ‘Natural killer cell mediated cytotoxicity’, which was significantly upregulated at 48 h is known to induce death receptor-mediated apoptosis, leading to caspase activation, mitochondrial dysfunction, and apoptosis [39]. The pathway of apoptosis from initial trigger to destruction of the cell can take hours to days [40], suggesting the mediators in this pathway could be influencing the expression patterns we observed. Clinical conditions that trigger tissue hypoxia are also known to promote inflammation and many immune cells are known to adapt to low oxygen conditions, such as in high altitude [41]. *Post-mortem* upregulation of immune and inflammatory responses may thus be triggered by tissue hypoxia associated with cessation of blood flow. By extension, immune status prior to slaughter may have implications during muscle to meat conversion as processes such as inflammation could affect several aspects of meat quality. For instance, inflammation could influence oxidative stability of membranes, water holding capacity, and maintenance of ion gradients, which could in turn influence calcium dependent protease activity, and onset and resolution of rigor. The finding of several disease related pathways upregulated at 72 h *post-mortem* may also be attributed to the fact that dying and dead cells, through apoptotic processes, stimulate inflammatory responses by acting through pathways that also underlie the pathogenesis of a number of those diseases [37].

There was no direct support in literature regarding upregulation of enriched terms related to ribosome and translation that we observed at 72 h. A study on *post-mortem* human blood transcriptome [3] reported that the majority of changes in gene expression occurred between 7 and 14 h after death, with thousands of genes showing DE equally in both directions relative to *pre-mortem* samples, followed by stabilization of the transcriptome between 14 and 24 h, and eventually deactivation. Therefore, we speculate that, *post-mortem* induced processes which normally start in the early hours of death may activate later in time for muscle stored in refrigerated conditions. Additionally, the specific characteristics of skeletal muscle that allow for some metabolic processes to continue anaerobically [42] may have contributed to processes continuing later than would be the case in other tissues.

Next, the biological processes and pathways enriched among sets of downregulated genes revealed an initial downregulation of oxidative phosphorylation at 6 h, followed by downregulation of “regulation of lipolysis in adipocytes” and signalling pathways at later time points. Oxidative phosphorylation occurs in mitochondria and oxygen is critical for their optimal function. The downregulation of oxidative phosphorylation, which coincided with our observation of a disproportionately high number of mitochondrial genes appearing as downregulated at early time points, may be explained by decreases in muscle oxygenation with loss of blood flow and transition of oxy- to deoxymyoglobin in *post-mortem* muscle. To our surprise, the initially downregulated oxidative phosphorylation process was subsequently upregulated at 72 h. This may be explained by several studies [43–46] suggesting mitochondria may be functioning in some capacity *post-mortem* in skeletal muscle, unrelated to coupled respiration, as evidenced by the accumulation of succinate after 24 h *post-mortem*, and the fact that succinate can increase mitochondria-mediated metmyoglobin reduction.

The disproportionately high number of non-coding genes appearing DE at all time points *post-mortem* may be of interest, not only to understand the roles they play in regulating processes after death but also in providing clues on how their disruption in life could involve disease states and departure from well-being. Long non-coding RNA are known to regulate gene expression at the epigenetic, transcriptional and post-transcriptional level, while small nucleolar RNA play essential roles in the nucleolytic processing of rRNAs and as guide RNAs in the post-transcriptional synthesis. Previous studies [7] indicate that epigenetic regulatory genes continue to modify chromatin structure in organismal death and thus change the accessibility of transcription factors to the promoter or enhancer regions. Our findings implicate the need for more research into the roles of mitochondrial genes and non-coding RNA in orchestrating processes after death, and by extension, to regulate processes during life.

## Conclusion

This study contributes to our current understanding of the muscle transcriptome and associated biological processes up to 72 h *post-mortem*, which coincides with the conversion of muscle to meat. Measurements of RNA quality and stability indicate alternative sampling regimes can be implemented on-line in the abattoir up to 72 h *post-mortem*. However, transcriptional activity changes over time, affecting different genes to different extents, and this must be considered when studying transcriptional activity and interpreting gene biomarkers related to desirable meat quality traits. Interestingly, at least for those biomarkers tested in this study, it may be valid to link their expression to meat quality traits for up to 24 h *post-mortem*. This study also highlights the importance of further research into the roles of mitochondrial and non-coding genes in the regulation or disruption of transcription in *post-mortem* muscle as well as in life, and how pre-slaughter immune status might influence *post-mortem* meat quality. Future directions of research to extend upon the findings in the current study would be to consider more tissue types, *pre-mortem* immune status and time points *post-mortem* to study how gene expression and associated biological processes progress in different tissues stored under refrigeration in abattoir conditions.

## Supporting information

S1 Fig Read mapping statistics by animal ID and sampling time. (PDF)

S2 Fig Principal Component Analysis (PCA) plot based on normalized read counts of all genes and samples from beef *LT* over time. (PDF)

S3 Fig Power analysis to determine the fold change level that can be reliably detected. (PDF)

S4 Fig Heat maps of DE genes in beef *LT* over time. Colour scale: red = upregulated; blue = downregulated.
(PDF)

S5 Fig Volcano plots of DE genes in beef *LT* over time. The genes are plotted and coloured based on false discovery rate (FDR) and fold change (FC): red if FDR<0.05, orange if absolute FC>1.5, and green if both. The 10 most significant DE genes (sorted by FDR) are labelled in cases where the gene symbols are known.
(PDF)

S1 Table Lists of DE genes in beef *LT* over time. (XLSX)

S2 Table Functional enrichment analyses of DE genes in beef *LT* over time using DAVID functional annotation tool. (XLSX)
