# Correction: Evaluation of RNA quality and functional transcriptome of beef *longissimus thoracis* over time *post-mortem*

**DOI:** 10.1371/journal.pone.0314423

**Published:** 2024-11-19

**Authors:** The PLOS ONE Staff

There are errors in the author special designation. The first, second, sixth, seventh and eighth authors should have been noted as contributing equally to this work and then the third, fourth and fifth authors.

The first two authors, Stephanie Lam and Arun Kommadath, should be noted as shared first co-authors on this work.

The publisher apologizes for the errors.
