# Supplementary figures and images for: Evaluation of RNA quality and functional transcriptome of beef longissimus thoracis over time post-mortem
Source: PLoS One. 2021 May 25;16(5):e0251868. doi: 10.1371/journal.pone.0251868 (PMC8148330; doi:10.1371/journal.pone.0251868)

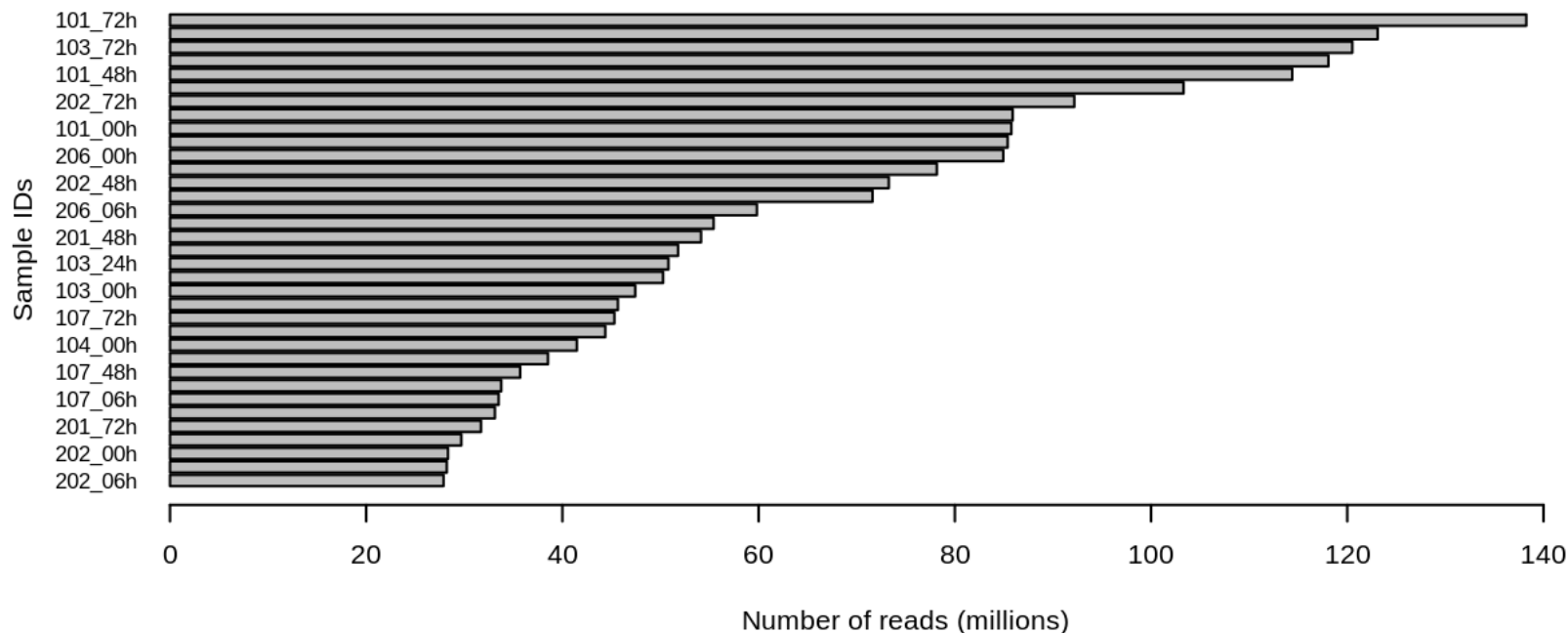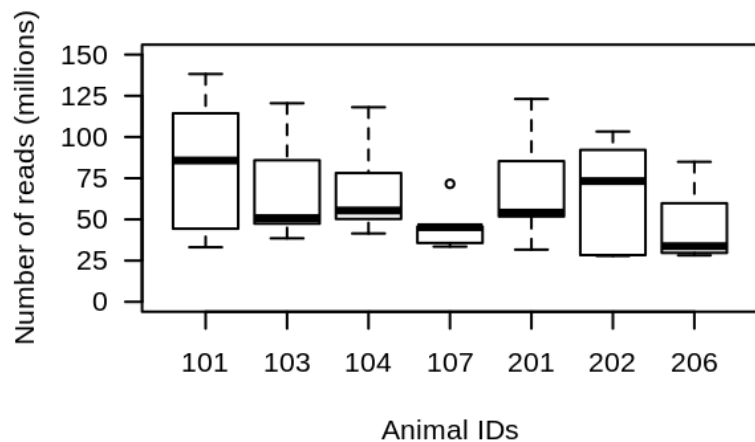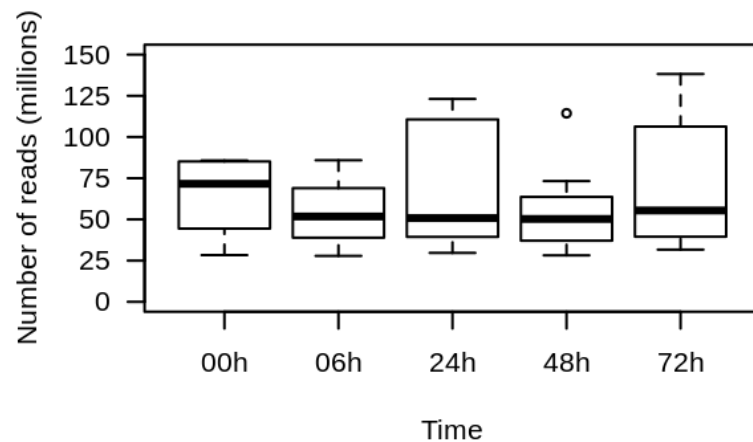

Supplement: S1 Fig — (PDF) [file pone.0251868.s001.pdf]

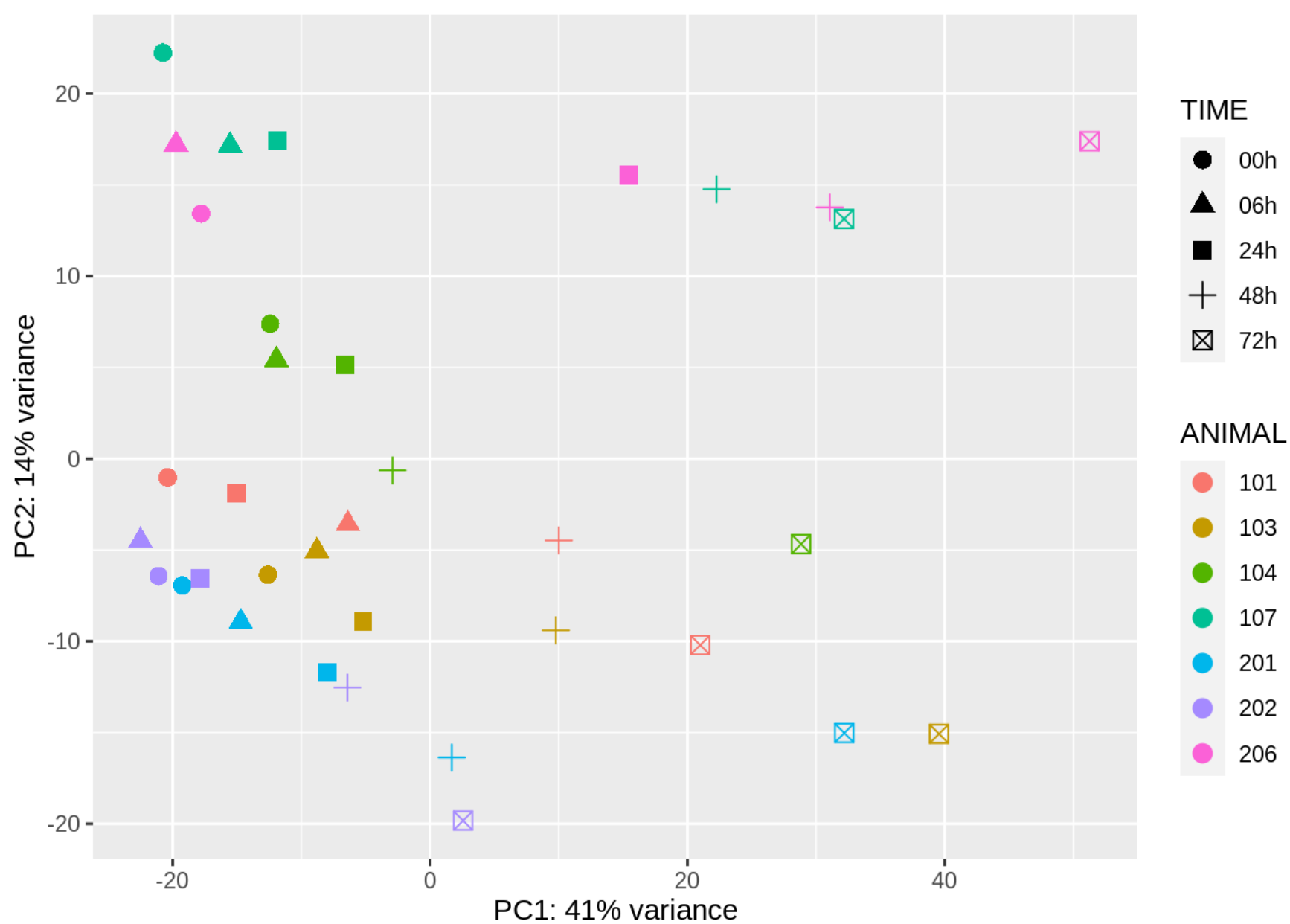

Supplement: S2 Fig — (PDF) [file pone.0251868.s002.pdf]

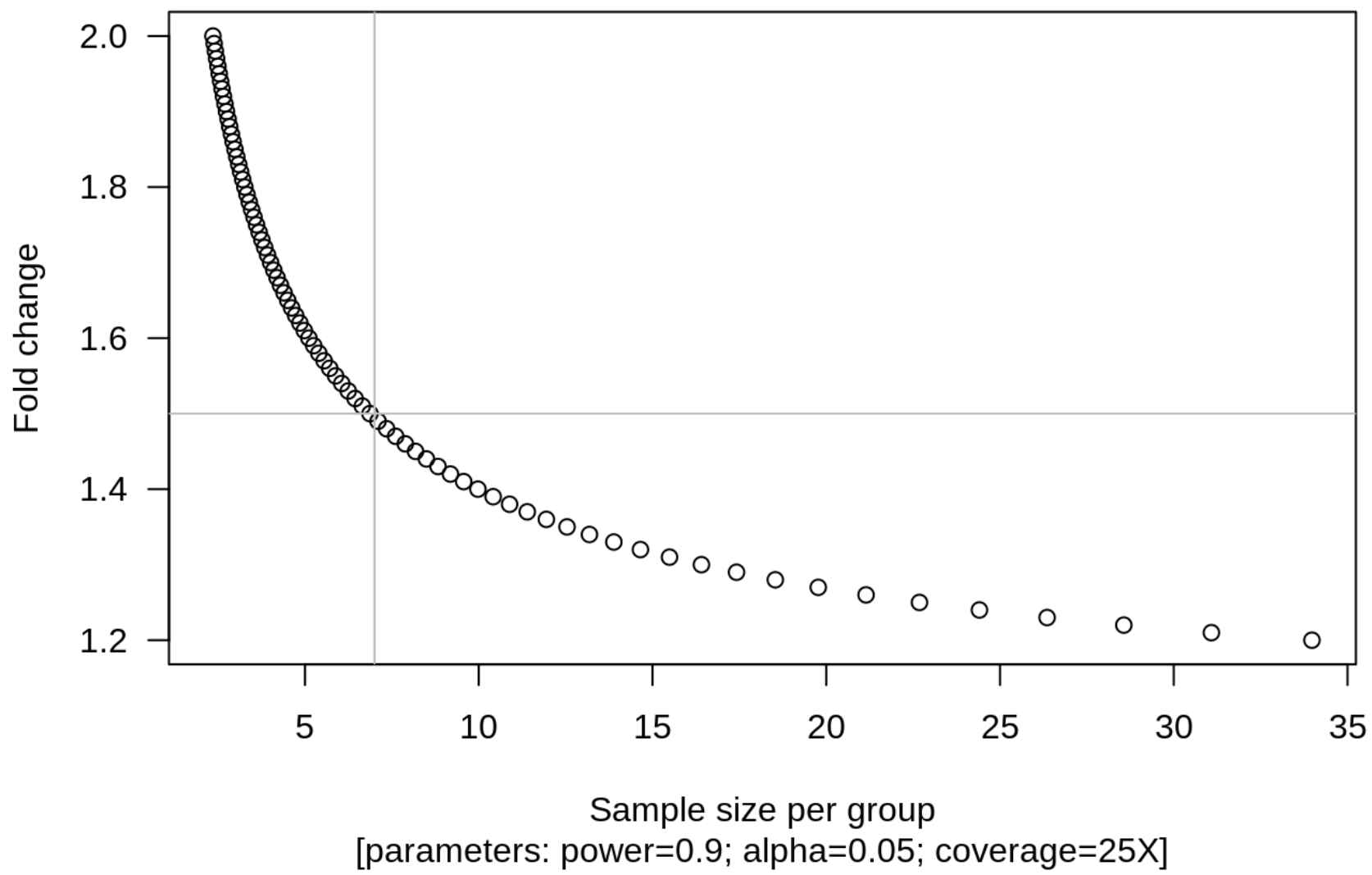

Supplement: S3 Fig — (PDF) [file pone.0251868.s003.pdf]

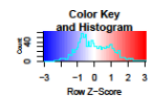

DE genes: 6 h vs. 0 h

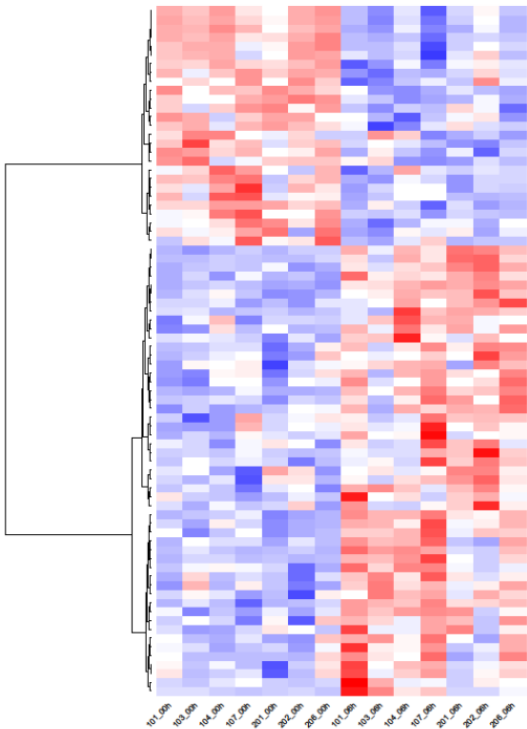

DE genes: 24 h vs. 0 h

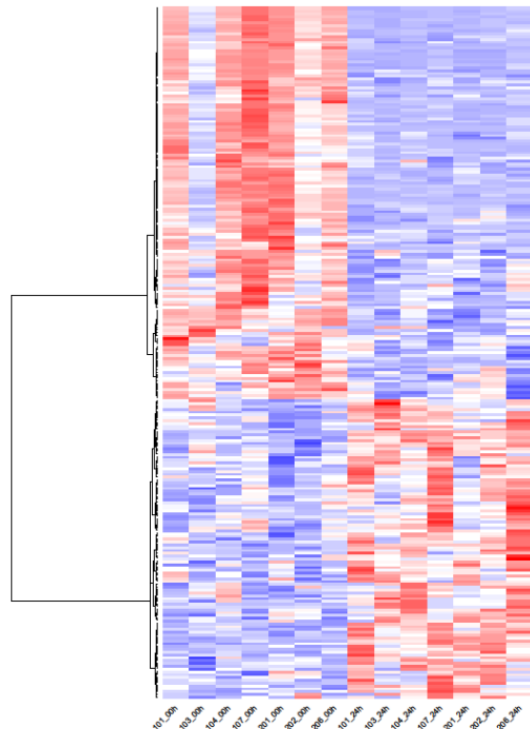

DE genes: 48 h vs. 0 h

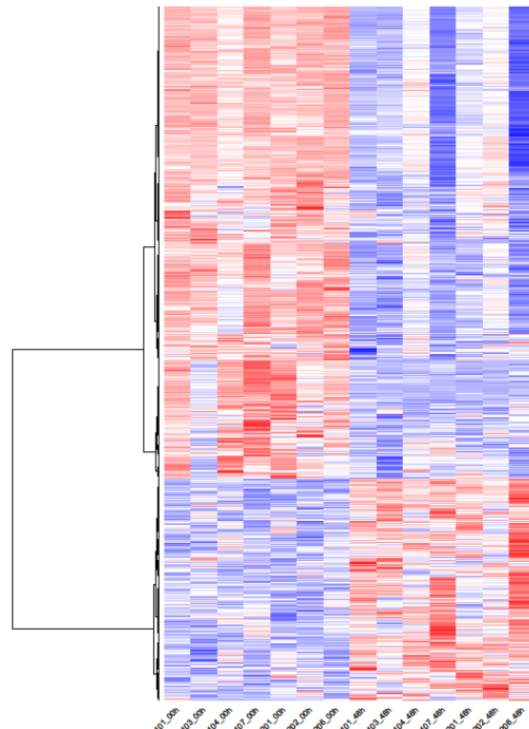

DE genes: 72 h vs. 0 h

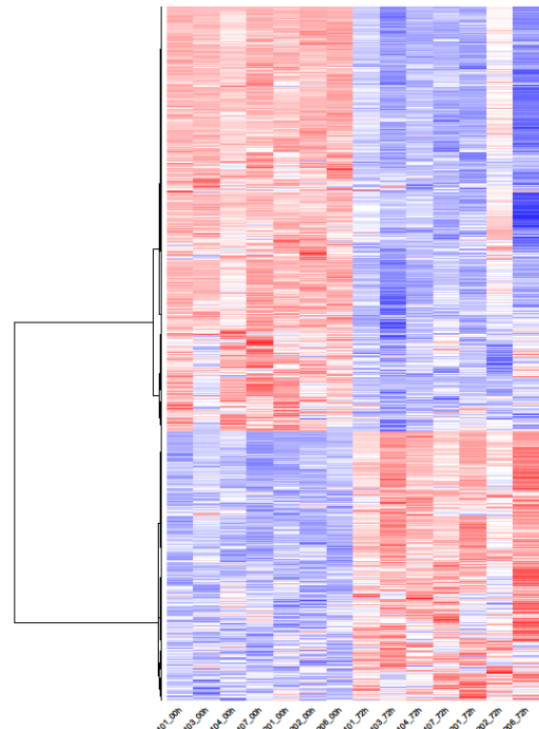

Supplement: S4 Fig — Colour scale: red = upregulated; blue = downregulated. (PDF) [file pone.0251868.s004.pdf]

DE genes: 6 h vs. 0 h

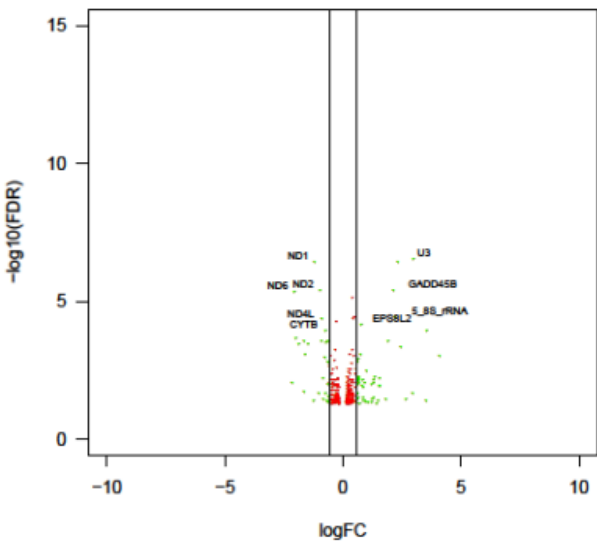

DE genes: 24 h vs. 0 h

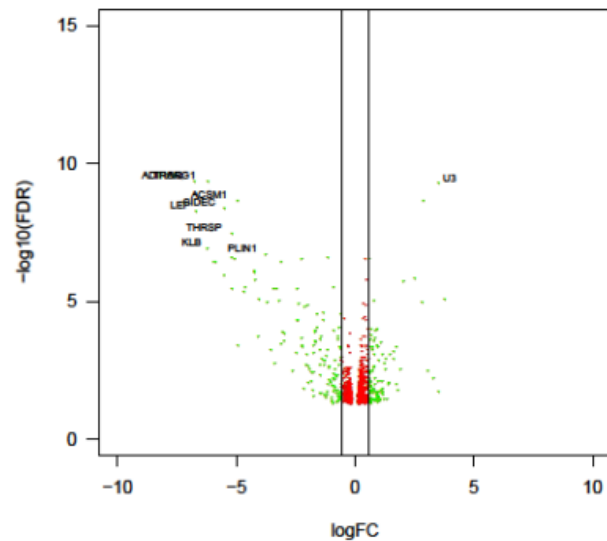

DE genes: 48 h vs. 0 h

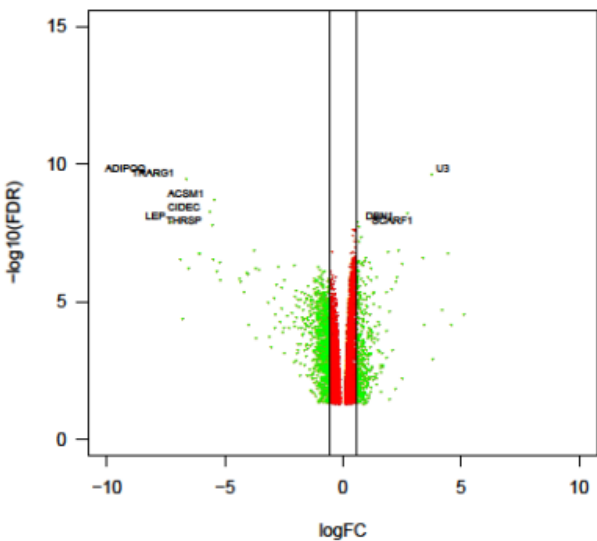

DE genes: 72 h vs. 0 h

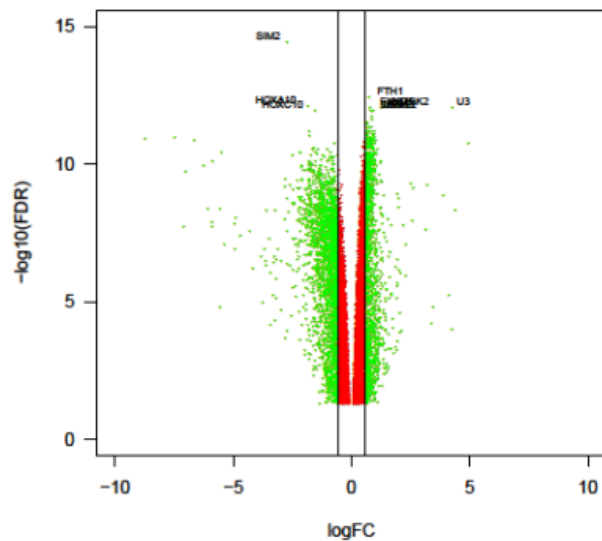

Supplement: S5 Fig — The genes are plotted and coloured based on false discovery rate (FDR) and fold change (FC): red if FDR<0.05, orange if absolute FC>1.5, and green if both. The 10 most significant DE genes (sorted by FDR) are labelled in cases where the gene symbols are known. (PDF) [file pone.0251868.s005.pdf]
